# AI-assisted MRI segmentation analysis of brain region volume alterations in Parkinson’s disease

**DOI:** 10.3389/fnhum.2025.1666556

**Published:** 2025-11-14

**Authors:** He Sui, Zhanhao Mo, Huiyan Luan, Weisha Yao, Meijun Wang, Lei Zhang

**Affiliations:** 1 Department of Radiology, China-Japan Union Hospital of Jilin University, Changchun, China; 2 Department of Neurology, Dongying People’s Hospital, Dongying, Shandong, China; 3 Department of Neurology, Yuncheng People’s Hospital, Yuncheng, Shanxi, China; 4 Department of Neurology, China-Japan Union Hospital of Jilin University, Changchun, China

**Keywords:** Parkinson’s disease, brain atrophy, structural magnetic resonance imaging, linear regression analysis, whole-brain region analysis

## Abstract

**Objectives:**

By employing deep learning-based automatic whole-brain region segmentation technology, we aim to investigate the cross-sectional associations between regional brain volumes and disease duration in patients with Parkinson’s disease (PD).

**Methods:**

A retrospective study design was implemented on 83 patients diagnosed with idiopathic PD who had complete clinical and imaging data. Cranial magnetic resonance images (MRI) were imported into the uAI platform for automated regional segmentation of brain tissue. Volumetric data from five major brain regions and 80 subregions were extracted to explore their potential associations with disease progression in PD patients. Statistical analysis was conducted using a multiple linear regression model within the framework of linear regression analysis, with statistical significance defined as *p* < 0.05.

**Results:**

Cross-sectional analysis revealed that in PD patients, volume ratios of multiple brain regions—including the bilateral precentral gyrus, right medial frontal gyrus, bilateral postcentral gyrus, bilateral superior and inferior parietal lobules, bilateral precuneus, right cuneus, right lingual gyrus, bilateral lateral occipital gyrus, and right globus pallidus—were negatively associated with disease duration (*p* < 0.05). In contrast, the right hippocampus, right inferior temporal gyrus, and left superior temporal gyrus showed positive correlations (*p* < 0.05). The combined volume ratios of these brain regions also decreased with longer disease duration (*p* < 0.05). Furthermore, absolute volume differences in the hippocampus, fusiform gyrus, isthmus of the cingulate gyrus, and cerebellar white matter increased as the disease progressed (*p* < 0.05).

**Conclusion:**

In PD patients, volume ratios and absolute volume differences in specific brain subregions associated with lateralized intracranial changes may serve as potential biomarkers for assessing brain tissue alterations during disease progression.

## 1 Introduction

Parkinson’s disease (PD) is the second most prevalent neurodegenerative disorder, characterized by cardinal motor symptoms including bradykinesia, tremor, and muscle rigidity. Nevertheless, substantial variability exists among patients in terms of clinical presentation, disease progression rates, and therapeutic responses (Jo et al., 2026). The clinical manifestations of PD are closely associated with disease duration: patients in the early stages predominantly exhibit motor symptoms, whereas those with longer disease courses frequently develop cognitive impairments and gait disturbances (Suzuki et al., 2022). This association suggests that the spatiotemporal patterns of atrophy across distinct brain regions may underlie the divergent clinical trajectories observed in PD in a cross-sectional perspective (Oh et al., 2024). This heterogeneity implies that distinct subgroups of patients may exhibit different patterns of brain network involvement. Structural magnetic resonance imaging (sMRI) offers a non-invasive means to longitudinally monitor *in vivo* atrophy in brain regions associated with PD pathology (Nguchu et al., 2025).

Previous cross-sectional studies have shown that brain regions such as the substantia nigra, striatum, hippocampus, and frontal lobe show significant volume loss in early PD, suggesting they are among the first areas affected. However, most of these studies involved small samples, limiting their statistical power and applicability to broader patient populations. In addition, many focused-on patients at similar disease stages, providing limited insight into how brain structure changes evolve across the full course of PD (Langley et al., 2025). Cross-sectional data only capture a single point in time and cannot reveal how individual brains change. Different brain regions may shrink at different rates—some rapidly in early stages and then stabilize, while others decline more slowly but continuously (Nyatega et al., 2022). Yet there are few systematic methods to quantify how these regional atrophy relates to disease duration. The current lack of understanding regarding the timing and patterns of neurodegeneration not only restricts insights into the underlying mechanisms of PD but also hinders accurate prediction of individual disease trajectories.

Traditional voxel-based morphometry (VBM) or manually defined region of interest (ROI) methods have identified reduced gray matter volume in key brain regions such as the hippocampus, temporal lobe, frontal lobe, and basal ganglia in patients with PD with mild cognitive impairment (PD-MCI) and Parkinson’s disease dementia (PDD), suggesting the potential role of these regions in disease-related cognitive decline (Wen et al., 2025). However, existing studies generally face the following limitations: Firstly, most studies rely on manual or semi-automated brain segmentation methods, which are not only time-consuming and labor-intensive but also have significant inter-operator variability, making it difficult to achieve consistent and reproducible segmentation results in large-scale samples. Such human factors may lead to measurement errors, affecting statistical power and the stability of conclusions. Secondly, most current studies adopt cross-sectional designs, which can only reflect structural changes at a single time point and fail to reveal the dynamic trajectory of atrophy over the course of the disease. Thirdly, existing methods typically employ coarse brain segmentation strategies, focusing only on a few large-scale brain regions while neglecting the simultaneous assessment of fine subcortical nuclei and cortical subregions. In fact, PD-related neurodegeneration often involves complex changes in brain networks, and only through systematic analysis at higher spatial resolution can the underlying pathophysiology be more comprehensively revealed.

This study applied deep learning techniques to perform automated segmentation of whole-brain MRI images from patients with PD, enabling precise quantification of volume parameters across 80 brain subregions and five key regions specifically associated with the disease. By examining the correlation between subregional volume proportions and disease duration, the study identified progressive patterns of brain atrophy in PD. Furthermore, the potential of these volumetric changes as imaging biomarkers for disease diagnosis and progression prediction was systematically evaluated, offering valuable data support and theoretical foundations for clinical assessment and decision-making.

## 2 Materials and methods

### 2.1 Participant sample

This retrospective study collected data from patients admitted to the Department of Neurology at China-Japan Union Hospital of Jilin University between January 2017 and December 2021. All patients included in the study were diagnosed with idiopathic PD and had complete clinical and imaging records. The study was approved by the Ethics Committee of China-Japan Union Hospital of Jilin University (No. 2023053014) and given its retrospective nature, the Institutional Review Board exempted the requirement for informed consent.

### 2.2 Inclusion and exclusion criteria

Inclusion criteria: Patients were included if they met the clinical diagnostic criteria for PD established by the Movement Disorder and PD Study Group of the Neurology Branch of the Chinese Medical Association.

Exclusion criteria: Patients with secondary parkinsonism, parkinsonism-plus syndromes, intracranial space-occupying lesions detected on routine MRI, a history of traumatic brain injury, or incomplete MRI imaging/data with artifacts were excluded.

### 2.3 Study methods

#### 2.3.1 Clinical data collection and grouping

(1) General clinical data were collected from PD patients who met the inclusion criteria, including demographic information such as gender, age, and disease duration.

(2) Grouping: Patients were categorized into two groups according to their disease duration: short disease duration (≤ median duration) and long disease duration (> median duration).

#### 2.3.2 MRI acquisition, quality control and multi-scanner harmonization

Magnetic resonance images data were retrospectively collected from the PACS systems across three campuses of China-Japan Union Hospital of Jilin University. A total of 10 3.0T MRI scanners were included, comprising Siemens Verio/Prisma (*n* = 3), United Imaging uMR 780/790 (*n* = 3), Philips Ingenia (*n* = 2), and GE Discovery MR750 (*n* = 2). Brain MRI scans from patients with Parkinson’s disease were imported into the uAI platform^1^ for automated whole-brain segmentation using deep learning. Segmentation was performed on axial T1-weighted images, and volume ratios of 80 brain regions—defined by the Automatic Anatomical Labeling atlas version 3 (AAL3)—were extracted, including five regions specifically implicated in Parkinson’s disease (Rolls et al., 2020). Prior to segmentation, all images underwent a “multi-vendor-robust” preprocessing pipeline designed to minimize variability in intensity, geometric distortion, and spatial resolution arising from differences in scanner manufacturer, magnetic field strength, and acquisition protocols. This ensured consistent, cross-scanner comparability and enabled reliable integration of multicenter data. The preprocessing workflow comprised seven critical steps: (1) automatic DICOM-to-NIfTI conversion; (2) joint correction of motion and geometric distortion; (3) intensity homogenization (bias field correction); (4) skull stripping with cavity filling; (5) resolution normalization and nonlinear registration to a study-specific template; (6) multi-site intensity harmonization; and (7) automated quality control with closed-loop feedback. The collected data were categorized into three research indicators: (1) the percentage of each brain (sub)region volume relative to the total brain volume (%), (2) the percentage of the combined volume of the left and right subregions relative to the total brain volume (%), and (3) the percentage of the absolute difference in volumes between the left and right subregions relative to the total brain volume (%). After automatic segmentation by the deep learning model, two senior radiologists (HS, ZH Mo) independently verified the accuracy of the subregion segmentations. The inter-rater agreement was assessed using Cohen’s kappa (κ = 0.89).

### 2.4 Statistical analysis

Quantitative data were tested for normality. Normally distributed data were expressed as mean ± standard deviation and compared using *t*-tests to evaluate significant differences. Non-normally distributed data were presented as median (interquartile range) and analyzed using rank-sum tests. Categorical data were summarized as proportions (%) or rates (%) and assessed using chi-square tests. All statistical analyses were conducted using multiple linear regression models to assess the association between disease duration and regional brain volume ratios. To account for multiple comparisons across 80 brain subregions, the false discovery rate (FDR) was controlled using the Benjamini–Hochberg procedure with a significance threshold of *q* < 0.05. Only findings that remained significant after FDR correction were considered statistically robust. Uncorrected *p*-values are additionally reported for exploratory purposes; however, these should be interpreted as indicative of potential associations and used to generate hypotheses rather than support definitive conclusions.


Y = α + β1 × age + β2 × gender + β3 × diseaseduration

In this equation, Y represents the proportion of brain region volume, α is the constant term, and β3 is the partial regression coefficient of disease course (denoted as Beta in the following text). When β3 is negative, it indicates a negative correlation between disease course and Y, meaning that as the disease progresses, the brain region volume gradually atrophies. When β3 is positive, it indicates a positive correlation, meaning that as the disease progresses, the brain region volume increases. Clinical data analysis was performed using SPSS 25.0 statistical software. A *p*-value less than 0.05 was considered statistically significant.

## 3 Results

### 3.1 Baseline data statistics of PD patients

A total of 379 PD patients diagnosed via imaging at China-Japan Union Hospital between January 2017 and December 2021 were enrolled. A total of 296 patients were excluded due to incomplete MRI data, unrecognized PD images by the uAI platform, or non-primary PD cases. The final cohort included 83 PD patients aged 46–83 years (mean age: 67.02 ± 9.74). Disease duration ranged from 0 to 16 years (median: 3 years), with short-duration cases averaging 1.59 ± 0.92 years and long-duration cases averaging 7.30 ± 2.95 years. There were 43 females (51.80%) and 40 males (48.10%). For baseline details, refer to Table 1.

**TABLE 1:** Baseline data of Parkinson’s disease (PD) patients.

Clinical indicator		Total	Course of disease		Gender	
			Short course	Long course	Male	Female
Age (year)		67.02 ± 9.74	67.49 ± 10.31	67.00 (62.00, 72.25)	69.50 (47.00, 67.00)	66.30 ± 9.09
Course of disease (year)		3.00 (1.5, 7)	1.59 ± 0.92	7.30 ± 2.95	2.75 (1.63, 5.75)	4.00 (1.00, 8.00)
Gender	Male	40 (48.10%)	24 (55.81%)	16 (40.00%)	–	–
	Female	43 (51.80%)	19 (44.19%)	24 (60.00%)	–	–

### 3.2 Baseline data statistics description of brain (subregion) volumes in PD patients

**FIGURE 1:**
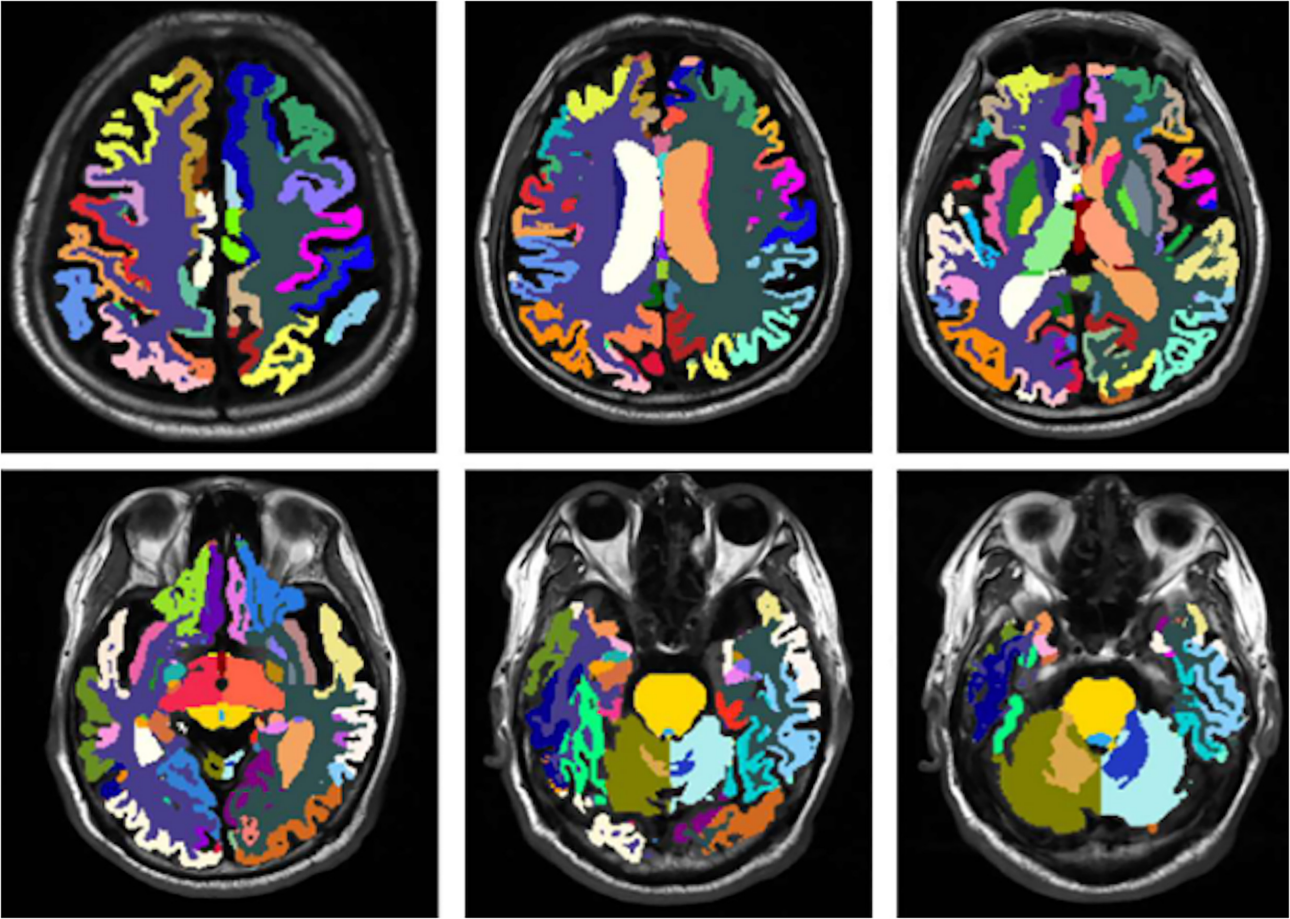
Schematic illustration of brain region segmentation. The uAI platform was adopted to automatically segment and visually display a total of 80 subregions in the whole brain.

**FIGURE 2:**
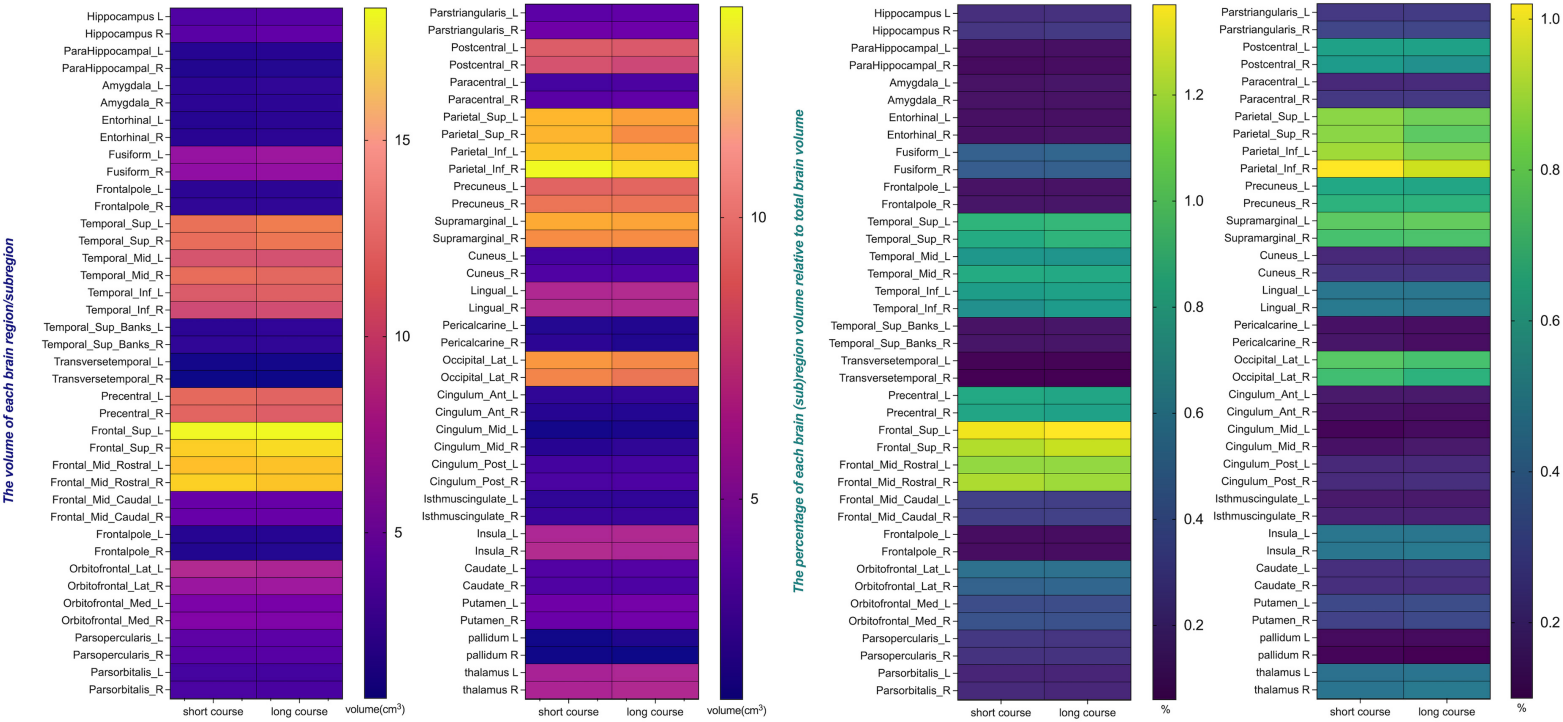
The volume and the percentage of each brain region/subregion volume relative to total brain volume. The heat map presents the volumes of individual brain subregions and their proportions of total brain volume in PD patients at different stages, providing essential data for further statistical analysis. Certain subregions show reduced volumes in long-term patients, indicating possible atrophy linked to disease progression.

**FIGURE 3:**
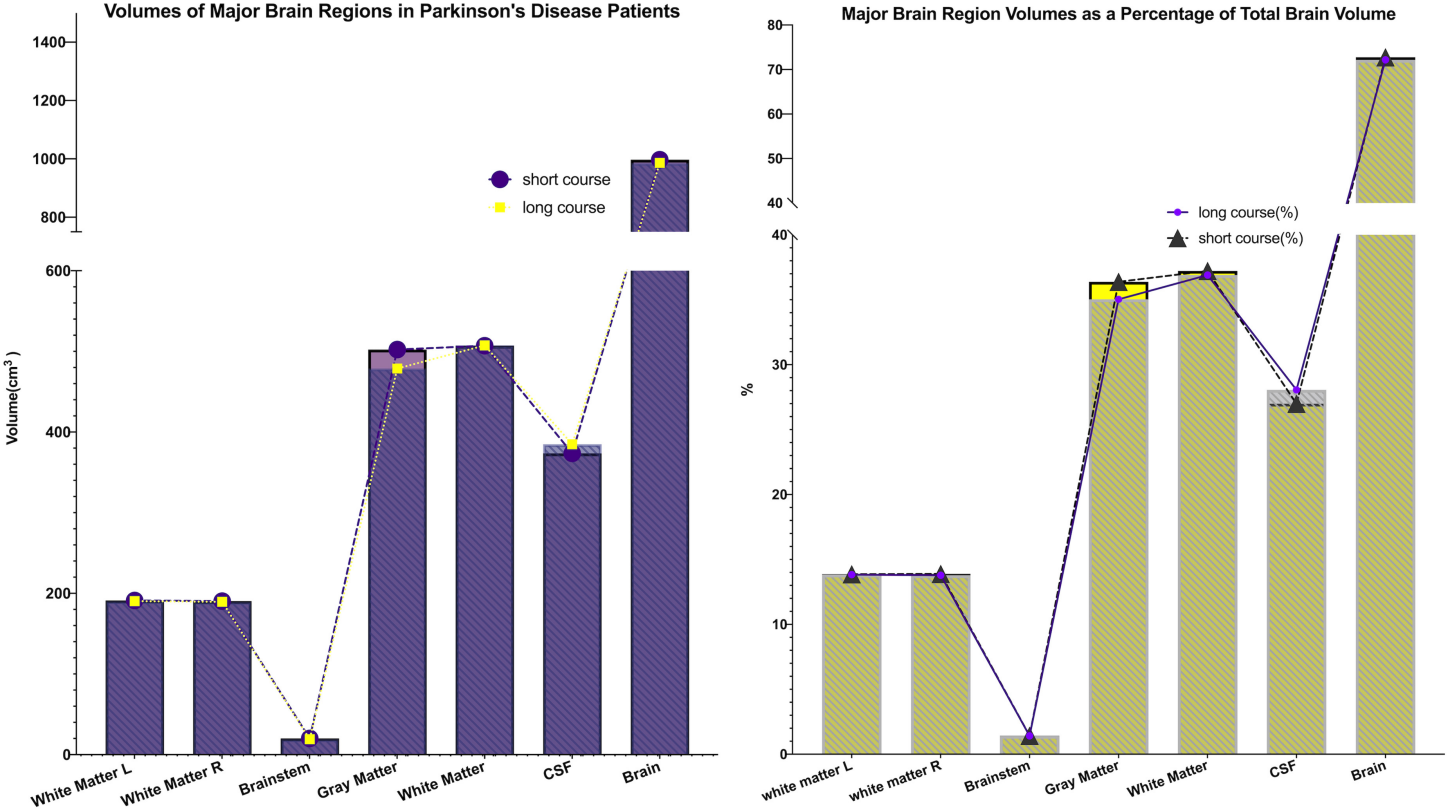
Major brain (sub)region volumes and their percentage of total brain volume. The volumes of five major brain regions (brainstem, gray matter, white matter, cerebrospinal fluid, and total brain tissue), as well as their proportions relative to total brain volume, were quantified. Both the volumes and proportional contributions of gray matter and white matter decreased progressively with longer disease duration, whereas the proportion of cerebrospinal fluid increased correspondingly. These findings suggest the presence of progressive parenchymal atrophy during disease progression.

**FIGURE 4:**
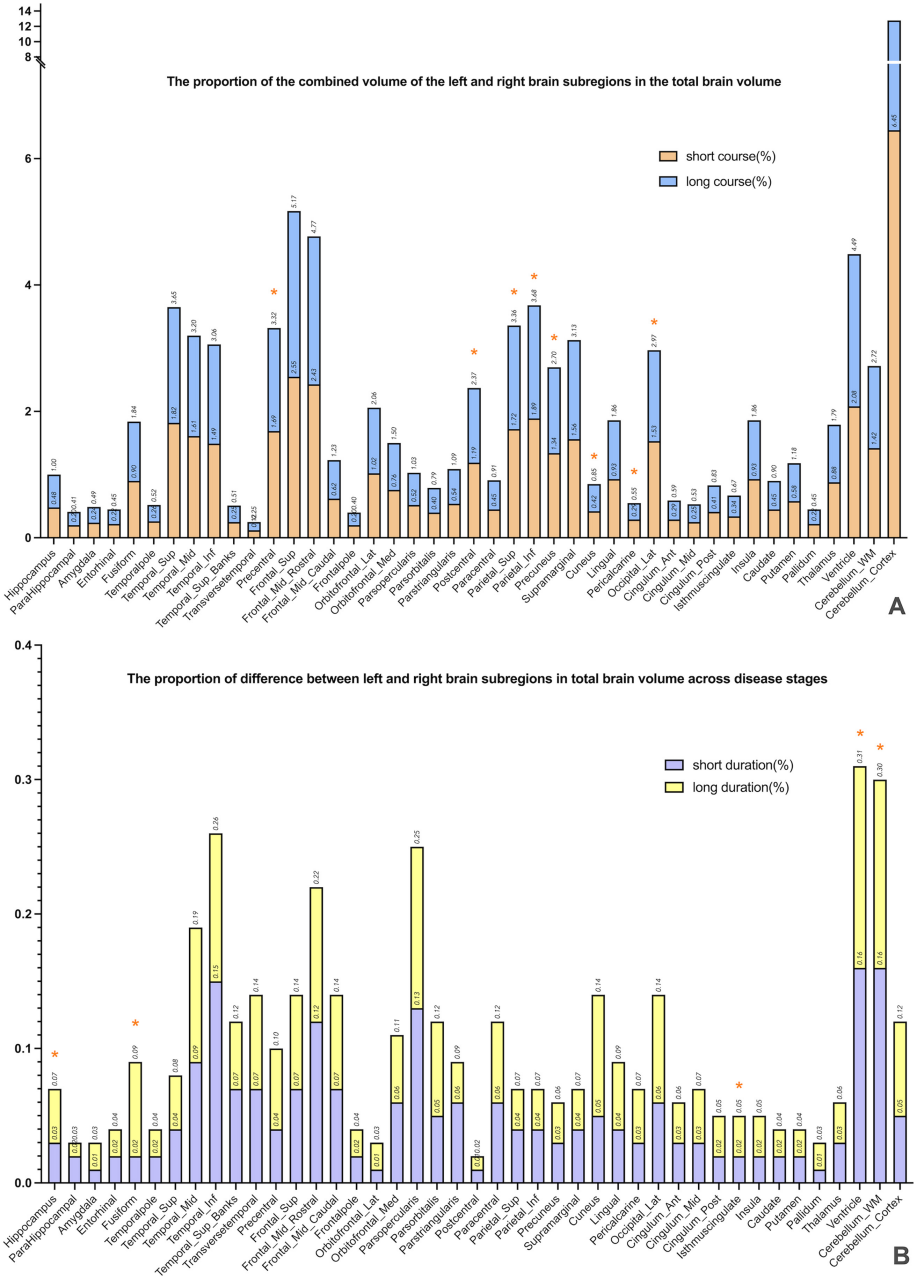
The combined volume and absolute difference between the left and right subregions, expressed as a percentage of total brain volume. **(A)** Presents a comparison of volume ratios of specific brain regions across different disease duration groups. By calculating the combined volume of left and right subregions relative to total brain volume, the study preliminarily identified brain regions potentially exhibiting progressive atrophy. The results revealed that in the long-duration group, the volume ratios of the bilateral precentral gyrus, superior parietal lobule, and precuneus—regions associated with motor-cognitive functions—were significantly lower than those in the short-duration group (* marked), indicating possible atrophy in these areas during disease progression. **(B)** Further investigates whether brain atrophy demonstrates lateralized progression. The analysis calculated the absolute volume differences between left and right subregions, expressed as a proportion of total brain volume. Findings showed greater left–right asymmetry in the hippocampus, fusiform gyrus, isthmus of the cingulate gyrus, and cerebellar white matter among patients with longer disease duration (* marked), suggesting a potential pattern of asymmetric atrophy in these regions.

This study included 83 PD patients. Volumes of 80 brain subregions and five major brain regions (brainstem, gray matter, white matter, cerebrospinal fluid, and brain tissue) were measured and are shown in Figure 1. Patients were divided into two groups by disease duration: short course (43 patients: 24 males, 19 females) and long course (40 patients: 16 males, 24 females). We calculated the mean ± SD or median (interquartile range) of each brain (subregion) volume (cm^3^) for both groups. Figures 2, 3 present the volume and proportion of each brain region relative to the total brain volume, along with the proportion of the combined bilateral brain region volumes to the total. The findings demonstrate that, among individuals in the long disease course group, the volume ratios of the bilateral precentral gyrus, superior parietal lobule, and precuneus (regions known to be involved in motor cognitive function) are slightly reduced in comparison to those observed in the short disease course group. Further analysis of the absolute volume differences and their proportions in the total brain volume between left and right brain subregions demonstrated that patients with a prolonged disease course exhibit greater left-right asymmetry in the hippocampus, fusiform gyrus, isthmus of the cingulate gyrus, and cerebellar white matter, indicating a potential asymmetrical atrophy pattern in these areas. Detailed information can be found in Figure 4.

### 3.3 Linear regression analysis of brain atrophy rate and disease duration in PD patients

#### 3.3.1 Linear regression analysis of brain subregion volumes and disease duration

Following FDR correction, significant associations between disease duration and regional brain volumes were observed in the following subregions: left precentral gyrus, left superior and right inferior parietal lobules, bilateral precuneus, left cuneus, right pericalcarine cortex, left lateral occipital gyrus, and right globus pallidus (FDR-corrected *q* < 0.05). Additionally, positive associations were identified in the left superior temporal gyrus (FDR-corrected *q* < 0.05). Full details of the FDR-adjusted *q*-values are presented in Table 2.

**TABLE 2:** Linear regression analysis of volumes of each brain subregion/whole brain and disease duration.

Subregions	Beta (95% CI)	*P*	*q*-value (FDR-adjusted)
Temporal_Inf_R	0.006 (0, 0.012)	0.038	0.646
Occipital_Lat_R	−0.008 (−0.015, −0.001)	0.032	0.272
Frontal_Mid_Rostral_R	−0.01 (−0.02, −0.001)	0.030	0.170
Postcentral_L	−0.007 (−0.013, −0.001)	0.029	0.123
Hippocampus_R	0.003 (0, 0.006)	0.025	0.085
Postcentral_R	−0.008 (−0.015, −0.001)	0.025	0.071
Precentral_R	−0.006 (−0.011, −0.001)	0.024	0.058
Parietal_Inf_R	−0.011 (−0.021, −0.002)	0.022	**0.047**
Precentral_L	−0.007 (−0.012, −0.001)	0.020	**0.038**
Precuneus_R	−0.007 (−0.014, −0.001)	0.020	**0.034**
Temporal_Sup_Banks_L	0.002 (0, 0.004)	0.016	**0.025**
Precuneus_L	−0.008 (−0.014, −0.002)	0.011	**0.016**
Pallidum_R	−0.002 (−0.004, −0.001)	0.011	**0.014**
Occipital_Lat_L	−0.009 (−0.015, −0.003)	0.006	**0.007**
Parietal_Sup_L	−0.015 (−0.024, −0.005)	0.003	**0.003**
Cuneus_L	−0.005 (−0.008, −0.002)	0.001	**0.001**
Pericalcarine_R	−0.004 (−0.007, −0.002)	0.001	**0.001**

L, left; R, right; Lat, lateral; Sup, superior; Mid, middle; Inf, inferior. Only results surviving false discovery rate (FDR) correction are bolded and considered statistically significant.

#### 3.3.2 Linear regression analysis of combined bilateral subregion volumes relative to total brain volume

We also analyzed the combined volume proportions of bilateral subregions relative to total brain volume. Significant negative correlations with disease duration were found for the precentral gyrus, postcentral gyrus, superior and inferior parietal lobules, precuneus, lingual gyrus, and lateral occipital gyrus (FDR-corrected *q* < 0.05). See Table 3 for details.

**TABLE 3:** Linear regression analysis of the sum of volumes of bilateral brain subregions relative to total brain volume and disease duration.

Subregions	Beta (95% CI)	*P*	*q*-value (FDR-adjusted)
Cuneus	−0.006 (−0.011, −0.001)	0.019	0.152
Postcentral	−0.015 (−0.026, −0.003)	0.011	**0.044**
Precentral	−0.013 (−0.022, −0.003)	0.009	**0.024**
Precuneus	−0.016 (−0.027, −0.004)	0.009	**0.018**
Parietal_Inf	−0.02 (−0.034, −0.005)	0.008	**0.013**
Occipital_Lat	−0.016 (−0.028, −0.005)	0.007	**0.009**
Pericalcarine	−0.006 (−0.011, −0.002)	0.004	**0.005**
Parietal_Sup	−0.035 (−0.052, −0.017)	<0.001	**0.000**

L, left; R, right; Lat, lateral; Sup, superior; Mid, middle; Inf, inferior. Only results surviving false discovery rate (FDR) correction are bolded and considered statistically significant.

#### 3.3.3 Linear regression analysis of left–right volume differences relative to total brain volume

Finally, we examined the absolute volume differences between left and right subregions relative to total brain volume. Significant positive correlations with disease duration were observed for the fusiform gyrus, cingulate isthmus (FDR-corrected *q* < 0.05). See Table 4 for details.

**TABLE 4:** Linear regression analysis of the absolute value of volume differences between left and right brain subregions relative to total brain volume and disease duration.

Subregions	Beta (95% CI)	*P*	*q*-value (FDR-adjusted)
Ventricle_Lat	0.016 (0.001, 0.031)	0.035	0.175
Hippocampus	0.002 (0, 0.004)	0.034	0.085
Cerebellum_WM	0.003 (0, 0.006)	0.031	0.052
Fusiform	0.004 (0.001, 0.007)	0.004	**0.005**
Cingulum	0.002 (0.001, 0.003)	<0.001	**0.000**

L, left; R, right; Lat, lateral; Sup, superior; Mid, middle; Inf, inferior. Only results surviving false discovery rate (FDR) correction are bolded and considered statistically significant.

The above linear regression analysis revealed significant associations between disease duration and region-specific volumetric alterations: (1) sensorimotor and parietal-occipital regions exhibited bilateral volume loss, whereas medial-temporal areas demonstrated relative preservation; (2) combined bilateral volume assessments corroborated this pattern of selective atrophy and sparing; (3) progressive left–right asymmetry was observed in the hippocampus, cingulate isthmus, and cerebellar white matter. These findings underscore the spatiotemporally heterogeneous nature of neurodegeneration in PD progression.

## 4 Discussion

Parkinson’s disease is characterized by a high incidence rate and significant disability and currently lacks reliable biomarkers for early diagnosis. In this study, we integrated the widely applied sMRI technique with a deep learning-based whole-brain automatic segmentation method to perform precise automated segmentation of whole-brain MR structures. Linear regression analysis was employed to investigate the association between disease progression and regional brain atrophy rates. The results demonstrate cross-sectional associations between longer disease duration and reduced volumes in the frontal, parietal, temporal, and occipital cortices, as well as in subcortical regions. Correlation analysis between imaging changes and clinical manifestations is crucial for identifying potential biomarkers in the preclinical stage of PD, which provides a critical foundation for the design and implementation of effective neuroprotective interventions.

### 4.1 Linear progression of cortical atrophy in PD patients

This study demonstrated that cortical atrophy in PD is extensive, affecting the frontal, temporal, parietal, and occipital lobes.

Firstly, in the frontal lobe, progressive atrophy was predominantly observed in the bilateral precentral gyrus and the anterior portion of the right middle frontal gyrus. Atrophy in these regions significantly impairs cognitive functions, particularly attention/executive function and memory encoding (Meng et al., 2023). Structural alterations in the frontal lobe are a critical factor contributing to executive dysfunction syndrome, a hallmark of early cognitive impairment in PD patients (Yang et al., 2024). This syndrome arises from diminished dopaminergic regulation within the prefrontal cortex-striatum circuit, leading to deficits in executive control (Iqbal et al., 2025). Pathological studies have also shown a positive correlation between the burden of Lewy bodies in the frontal cortex and cognitive impairment in PD (Borghammer et al., 2024; Vidyadhara et al., 2024, 2025).

Secondly, in the parietal lobe, progressive atrophy was primarily detected in the bilateral precuneus, postcentral gyrus, superior parietal lobule, and inferior parietal lobule. The precuneus serves as a pivotal node for information integration within the parietal network (Baun et al., 2025). Positron emission tomography (PET) studies have revealed reduced metabolism in the precuneus and other posterior parietal regions in PD patients, correlating with declines in memory and executive function in PD patients without dementia (Cao et al., 2024; Yeager et al., 2024). The superior parietal lobule, inferior parietal lobule, and precuneus are interconnected in neural networks and play essential roles in integrated executive function processing (Fernandez-Del-Olmo et al., 2023; Mano et al., 2022). Morphological changes in these regions may independently contribute to declines in executive function or interact pathophysiological with basal ganglia dysfunction (Guo et al., 2022; Oikonomou and Jost, 2022).

Finally, in the occipital lobe, progressive atrophy was mainly observed in the left cuneus, right fusiform gyrus, and bilateral lateral occipital gyrus. Meanwhile, the parietal lobe (including the precuneus, superior parietal lobule, and inferior parietal lobule) has been consistently linked to executive dysfunction and impaired visuospatial ability in longitudinal neuropsychological studies of PD (Li et al., 2018). Executive function involves key cognitive processes such as planning, decision-making, attention regulation, and multitasking, all of which are frequently compromised in patients with PD (Gul and Yousaf, 2021). Visuospatial decline manifests as reduced capacity to interpret spatial relationships, judge directions, and integrate visual information. This impairment not only affects daily activities—such as driving and navigation—but may also contribute to gait disturbances and an increased risk of falls (Petkus et al., 2024). Therefore, neurodegenerative changes in the parietal lobe likely play a central role in the progression of cognitive impairment in PD and warrant prioritization in future clinical assessments and intervention strategies. The occipital lobe serves as the primary visual cortex center. Damage to this region not only causes visual impairment but also contributes to symptoms such as memory deficits and motor perception disorders, which are frequently observed in PD patients (Nyatega et al., 2022).

It is important to note that this study applied FDR correction to address the issue of multiple comparisons across numerous brain subregions. While this method effectively reduces the risk of Type I errors, it simultaneously raises the threshold for statistical significance, thereby increasing the likelihood of false negatives. As a result, certain regional volume changes that did not survive FDR correction may still reflect biologically meaningful patterns. These findings, though not statistically significant after correction, could represent potential neuroanatomical trends and warrant further investigation in future studies with larger sample sizes or through meta-analytic approaches.

### 4.2 Linear progression of subcortical gray matter atrophy in PD patients

This study analyzed both the linear progression of cortical volume changes and the alterations in the main nuclei of the basal ganglia. Progressive atrophy was observed in the right globus pallidus. Previous studies have shown that low-frequency (4–6 Hz) resting tremor, a hallmark symptom of PD, is associated with pallidal atrophy and dopamine deficiency (Asendorf et al., 2024). Unlike other PD symptoms, resting tremor is linked not only to pathophysiological changes in the basal ganglia but also to pathological alterations in the motor areas of the cerebellar-thalamocortical circuit. Tremor activity originates in the internal segment of the globus pallidus (GPi) and subsequently spreads through the motor cortex to the cerebellar-thalamocortical circuit. These findings support the “dimmer switch” hypothesis, which suggests that GPi modulates the cerebellar-thalamocortical circuit, acting as a control mechanism for tremor intensity (Sadeghi et al., 2024; Zhong et al., 2025). This research has significant therapeutic implications for PD patients, indicating that resting tremor could be mitigated by preventing the amplification of tremor signals originating from the basal ganglia within the cerebellar-thalamocortical circuit (Wang et al., 2025).

### 4.3 Lateralized progression of brain atrophy in PD patients

This study investigated the linear relationship between disease duration and the absolute volume differences between left and right brain subregions as a proportion of total brain volume. The results demonstrated that the volume differences in subregions such as the hippocampus, fusiform gyrus, isthmus of the cingulate gyrus, lateral ventricle, and cerebellar white matter increased with disease progression. Additionally, the volume proportions of the anterior portion of the right middle frontal gyrus and the right globus pallidus were negatively correlated with disease duration, indicating progressive atrophy. However, when considering the sum of the bilateral volumes of these regions as a proportion of total brain volume, no significant changes over time were observed, further supporting the lateralized nature of atrophy.

One of the defining characteristics of PD is its lateralized onset. Accumulating pathological and imaging evidence has demonstrated that brain changes in PD exhibit hemispheric asymmetry, a finding that is further supported by the present study (Massaranduba et al., 2025). Previous research indicates that the right hemisphere is typically more affected in the early stages, with cortical involvement becoming increasingly bilateral as the disease progresses. However, findings regarding the asymmetry of brain region involvement remain inconsistent: some studies report greater right-hemisphere involvement, while others highlight more pronounced left-hemisphere effects (Yuan et al., 2024). The underlying mechanisms driving this hemispheric asymmetry are not yet understood. Due to the absence of clinical laterality data in this study, it remains unclear whether the observed lateral atrophy corresponds to the laterality of motor or cognitive symptoms. To date, no conclusive evidence links this asymmetry to cerebral dominance or the hemisphere with more severe symptom expression. Further prospective investigations are warranted to clarify these relationships. Furthermore, this study observed an intriguing phenomenon: the proportions of the right hippocampus, right inferior temporal gyrus, and left superior temporal gyrus increased with disease progression. This may be attributed to the dependent variable being the proportion of brain region volume relative to total brain volume. When the overall brain atrophy rate exceeds that of specific subregions, it can result in an apparent increase in the proportion of these subregions. Whether this “increase” reflects a brain reserve factor that protects cognitively unimpaired regions or is merely an artifact of global brain atrophy requires further investigation through longitudinal studies.

In previous studies on PD, deep learning techniques have primarily been applied to specific MRI sequences, such as Quantitative Susceptibility Mapping (QSM) and T1 mapping, for the detection of abnormal signals, segmentation of distinct brain nuclei, and delineation of extracellular spaces (Li et al., 2024; Telepatil and Vaddin, 2025). However, these approaches have not yet achieved comprehensive whole-brain structural segmentation using conventional MRI sequences, potentially missing certain brain regions associated with PD. This study applies a deep learning segmentation model to standard MRI sequences, thereby expanding the scope of application and significantly improving the method’s generalizability, making it more suitable for widespread clinical adoption. The deep learning-based whole-brain multi-region segmentation model used in this study demonstrates high segmentation accuracy and strong reproducibility. Although the study was conducted at a single center, the image data were collected from three geographically diverse medical institutions and acquired using 10 MRI scanners from four different manufacturers. This variability in data sources enhances the model’s adaptability across different populations and disease stages to a considerable extent. Given that deep learning models are often considered “black boxes” due to their opaque decision-making processes, we aim to improve model interpretability by incorporating attention mechanism-based architectures in future work. This will allow for a more in-depth investigation of the key features and underlying decision-making criteria used by the model during the segmentation process.

This study reveals that certain brain regions exhibit a slight increase in volume as Parkinson’s disease progresses—a finding that appears counterintuitive given the neurodegenerative nature of the condition. This paradoxical structural change may be accounted for by two distinct mechanisms. First, the concept of “brain reserve” suggests that individuals with larger premorbid hippocampal volumes possess a greater structural buffer, enabling them to tolerate ongoing neuropathology longer before atrophy becomes detectable on imaging. This decoupling between underlying pathology and observable structural change has been well documented in Alzheimer’s disease research, supporting the role of brain reserve in delaying the clinical and radiological expression of neurodegeneration (Coulter et al., 2024). Second, “compensatory hypertrophy” may contribute to transient regional expansion through reactive processes such as glial activation, microvascular proliferation, or vascular remodeling (Pombero et al., 2023; Zhang et al., 2025). While these changes can manifest on MRI as stable or even increased tissue volume, they do not necessarily reflect functional recovery; instead, they may signal early neuroinflammatory activity or blood-brain barrier disruption. Although potentially beneficial in the short term by supporting neural compensation, such adaptations may ultimately destabilize the local microenvironment and accelerate long-term degeneration.

Based on cross-sectional data and existing metabolic and pathway evidence, we propose a three-stage model of neurodegeneration in Parkinson’s disease. The first stage involves focal atrophy, marked by unilateral degeneration of the sensorimotor cortex, superior parietal lobule, and globus pallidus, aligning with the “dimmer switch” tremor circuit. The second stage features network-based spread, characterized by progressive atrophy of bilateral parieto-occipital regions—including the precuneus and lateral occipital cortex—reflecting α-synuclein propagation along cortical connections, while the medial temporal lobe remains relatively preserved, resulting in an “inverted” volumetric profile. The third stage is asymmetric degeneration, with increasing left-right divergence in limbic structures (e.g., hippocampus, isthmus cingulate) and cerebellar white matter, consistent with trans-synaptic progression through the cerebello-thalamo-cortical and dopaminergic reward circuits. Within this framework, apparent “volume increase” reflects slower atrophy in regions with higher reserve capacity, not actual tissue growth; pronounced hemispheric asymmetry underscores the need for hemisphere-specific biomarkers. While this model remains hypothetical, it offers a mechanistically grounded, testable framework for future longitudinal and multimodal investigations.

### 4.4 Limitations

This study has several limitations that warrant consideration. First, the relatively small sample size may compromise statistical power and limit the generalizability of the observed findings. Larger-scale studies in the future would enhance the reliability of the identified atrophy patterns and enable more refined subgroup analyses based on disease duration and severity. Second, due to the retrospective data collection, Unified PD rating scale (UPDRS) scores and medication records were often incomplete. Therefore, disease severity and medication status were not included as covariates in this analysis. Future prospective studies will collect standardized UPDRS-III and Levodopa Equivalent Daily Dose data to better validate the relationship between disease duration and brain atrophy. Third, although we included multiple relevant covariates and excluded major confounding conditions, residual confounding cannot be entirely ruled out. Prospective studies with systematic collection of variables such as anti-Parkinson’s medication use and cardiovascular metabolic comorbidities are needed to more thoroughly validate the current findings. Fourth, while the present study employed a cross-sectional design, the observed association between disease duration and regional brain volume aligns with evidence from longitudinal investigations. Nevertheless, longitudinal imaging follow-up is necessary to confirm the dynamic trajectory of these neurostructural changes over time.

## 5 Conclusion

This study applied deep learning-based fully automated segmentation techniques to widely utilized sMRI data, performing a comprehensive whole-brain regional segmentation analysis. The results demonstrate that, as PD progresses, brain volume alterations exhibit cross-sectional associations with disease duration and show a distinct pattern of hemispheric lateralization. These methodological approaches offer a valuable framework for *in vivo* investigation of multiple aspects of PD pathology, with potential use as early diagnostic biomarkers, tools for longitudinal monitoring, and objective endpoints for assessing treatment efficacy in future clinical trials.

## Data Availability

The raw data supporting the conclusions of this article will be made available by the authors, without undue reservation.

## Footnotes

1
https://urp.unitedimaging.com/#/
